# Qualitative Study of Health Care Professional Perspectives on Recruiting Minority Patients in Cancer Trials

**DOI:** 10.1089/heq.2024.0184

**Published:** 2025-01-20

**Authors:** Katharine A. Price, Rahma Warsame, Elhadji A. Toure, Molly O’Shea, Yong-Hun Kim, Sara A. Ellingson, Joselle M. Cook, Gladys B. Asiedu

**Affiliations:** ^1^ Division of Medical Oncology, Mayo Clinic, Rochester, Minnesota, USA.; ^2^ Department of Medicine, Division of Hematology, Mayo Clinic, Rochester, Minnesota, USA.; ^3^ Department of Otolaryngology-Head and Neck Surgery, Undergraduate Summer Research Intern, Mayo Clinic, Rochester, Minnesota, USA.; ^4^ Alix Mayo Medical School, Rochester, Minnesota, USA.; ^5^ Mayo Clinic Robert D. and Patricia E. Kern Center for the Science of Health Care Delivery, Rochester, Minnesota, USA.

**Keywords:** clinical trial, health equity, ethnic and racial minority groups, healthcare professionals, research participant recruitment

## Abstract

**Background:**

Underrepresented minority (URM) patients enroll in cancer clinical trials at low rates. We studied the perspectives of health care professionals to better understand the challenges and potential facilitators of recruiting URM patients into clinical trials.

**Methods:**

A purposeful sampling approach was utilized to recruit health care professionals and clinical research staff who recruited and cared for URM patients in a therapeutic cancer clinical trial at any Mayo Clinic site, from July 2018 through October 2021.Data were gathered using a semistructured qualitative interviews. Participants were interviewed about the challenges of recruiting URM patients into cancer clinical trials and possible strategies for improving URM patient enrollment. Data were analyzed using a thematic analysis.

**Results:**

Of the key themes that emerged from participant interviews (*n* = 28), perceived barriers to recruiting URM patients into clinical trials included lack of workforce diversity, underutilization of patient navigators, ineffective community outreach and patient recruitment, restricted access to diverse patient populations, and restrictive clinical trial eligibility criteria. Other barriers reported were lack of insurance and access to care and transportation, low socioeconomic status, and mistrust of health care and research. Strategies suggested for improving the recruitment of URM patients into clinical trials included: diversifying and adding research staff, increasing and improving community outreach and advocacy, partnering with clinics closer to patients, increasing monetary and logistical support for patient participation, creating opportunities that build and enhance trust, and systematically examining and modifying clinical trial eligibility criteria.

**Conclusions:**

Our findings reveal barriers that have limited URM patient inclusion in cancer clinical trials and highlight strategies to overcoming these barriers.

## Introduction 

Clinical trials help advance cancer research and treatment, potentially leading to more effective therapies and improved patient outcomes. Some data indicate that patients participating in clinical trials can have better survival rates than patients receiving standard therapies.^1^ However, in therapeutic cancer clinical trials, the low recruitment rates of underrepresented minority (URM) patients make the opportunity for improved outcomes through research participation inequitable for URM patients. This lack of diverse representation in clinical trials limits not only the universality of the trial results but also the application of trial findings, which widens health care disparities.

Despite efforts at the national and institutional levels to increase URM patient participation in clinical trials, URM patients remain underrepresented in cancer trials. This underrepresentation may be attributable to multiple factors, including personal, systemic, and logistical barriers that hinder inclusive clinical trial participation. Personal barriers include demographic characteristics, socioeconomic status, and informational and perceptual barriers that may impede patient comprehension and willingness to participate in trials.^2,3^ Furthermore, inadequate compensation for trial participation can dampen the enthusiasm of potential URM participants.^4^ Systemic barriers include aspects of clinical trial design that inadvertently exclude URM communities.^2^ For example, inclusion and exclusion criteria may not consider the limited health care access and insurance status of some URM patients. In addition, historical injustices in medical research that have resulted in a legacy of distrust have continued to affect participation rates among URM patients.^3,5^ Finally, logistical barriers for recruitment and retention of URM patients in clinical trials include patient relocation, personal obligations, loss to follow-up, and nonadherence to study requirements.^4^

To date, most research on URM patient accrual in clinical trials has focused on the perspective of patients, with more limited published data on the perspective of health care professionals.^3,6,7^ However, studying the unique front-line perspective of health care professionals is critical for comprehensively understanding the obstacles to inclusive clinical trial recruitment and for devising effective strategies to overcome them.^4^ Because health care professionals are often the primary communicators of trial information, they can provide insight into how comprehension, trust, and accessibility issues may affect URM patient recruitment. Health care professionals can also identify potential institutional or systemic practices, such as scheduling issues and language barriers, that may inadvertently hinder the inclusion of URM patients in clinical trials. In order to understand the specific barriers of minority accrual into trials within our institution, we interviewed different types of health care professionals and clinical research staff to better understand the barriers and facilitators of recruiting URM patients in cancer clinical trials. Our findings may inform future institutional strategies for increasing URM patient accrual.

## Methods

This was a multi-site single institution study conducted at the main Mayo Clinic campuses in Minnesota, Florida, and Arizona. Mayo Clinic is a National Cancer Institute designated comprehensive cancer center serving a diverse range of patients across geographic regions. The study was approved by the Mayo Clinic Institutional Review Board (# 21-001328). A purposeful sampling^8,9^ was utilized to identify our study population. Participants were selected based on their involvement with clinical trials. By this approach, we searched our electronic health record and institutional clinical trial databases for URM patients treated as part of an interventional therapeutic cancer clinical trial at Mayo Clinic in Minnesota, Arizona, or Florida from July 2018 through October 2021. We then identified the health care professionals and clinical research staff who enrolled and/or followed up URM patients during the trial and invited them via email to participate in interviews. URM patients were defined as Black or African American, Native Hawaiian or Pacific Islander, Native American or Alaska native, more than 1 racial and ethnic group, or Hispanic or LatinX. Health care professionals and clinical research staff who agreed to be interviewed were scheduled for a virtual interview. Reminders were sent one day before interview dates. A second email invitations were sent to those who did not respond. All data were gathered via zoom.

An interview guide was developed for the semi structured interviews. A trained qualitative researcher (G.B.A.) used the interview guide (Supplementary Data) to conduct interviews with all health care professionals and clinical research staff who provided informed consent. Data were collected for the following characteristics of study participants: self-reported gender, self-reported race and ethnicity (Asian, Black, Hispanic, White), professional role (physician, scientist [PhD], physician assistant, nurse navigator [i.e., a nurse who helps patients navigate the health care system], nurse supervisor, clinical research coordinator), and academic rank (professor, assistant professor, associate professor, no academic rank). The researcher explored the experiences of health care professionals and clinical research staff with enrolling and treating URM patients in therapeutic cancer clinical trials. Questions addressed aspects of care that went well, aspects of the process that could be improved, perceptions of barriers faced by URM patients and how they were overcome (if they were overcome), and recommendations for improving the overall trial process (Supplementary Data). Additionally, the researcher inquired about the resources potentially necessary to recruit URM patients to clinical trials. The study aimed to achieve data comprehensiveness rather than saturation and sample size, therefore we chose to fully interview all respondents who agreed to participate.

Qualitative interviews were recorded, transcribed verbatim, deidentified, checked for accuracy, and entered into Nvivo qualitative analysis software (version 12, QSR International Pty Ltd) for data management and analysis. This software program allowed for coding, categorizing, and synthesizing of data for specific ideas, themes, and topics of interest, and for generating reports at the end of the coding process. Data were analyzed using thematic analysis as described by Braun et al.^10–12^ This process involves rereading the interview transcripts to familiarize with the data. Initial codes were identified, and a coding guidance was developed and applied across all the transcripts. Codes were then categorized into themes. Emerging themes were discussed among study team. This process is a systematic approach to coding and analyzing qualitative data, ensuring that important themes are identified and validated through team discussions. Consistent with accepted qualitative research methods, data reliability was enhanced by ensuring data triangulation with data gathered from different groups of participants.^13^ Investigator triangulation was achieved by involving study team members from multidisciplinary fields.

## Results

From October through December 2021, 28 health care professionals and clinical research staff (15 [54%] female, 13 [46%] male) participated in semistructured interviews about their experience with URM patients in cancer clinical trials. Table 1 shows study participant characteristics including race and ethnicity, professional role, and academic rank. Most participants were physicians and White. Interview duration was approximately 30 min. Key themes that emerged from the interviews on challenges and barriers to URM patient recruitment are summarized in Table 2 and detailed in the Supplementary Table S1.

**Table 1.** Characteristics of Interviewed Health Care Professionals and Clinical Research Staff

Characteristic	No. of persons (%) (*n* = 28)
Gender	
Male	13 (46)
Female	15 (54)
Race and ethnicity	
Asian	3 (11)
Black	1 (4)
Hispanic	2 (7)
White	22 (79)
Professional role	
Physician	17 (61)
Scientist (PhD)	1 (4)
Physician assistant	2 (7)
Nurse navigator	2 (7)
Nurse supervisor	1 (4)
Clinical research coordinator	5 (18)
Academic rank	
Professor	9 (32)
Associate professor	5 (18)
Assistant professor	5 (18)
No academic rank	9 (32)

**Table 2.** Summary of Barriers and Strategies to Recruiting Underrepresented Minority Patients to Cancer Clinical Trials

Barrier or challenge	Strategies or solutions
Logistical barriers	
Lack of workforce diversity	Accelerate efforts to address workforce diversity gaps Strategically hire diverse research staff
Underutilization of patient navigators	Increase staffing and create designated roles for patient navigators
Restricted access to diverse patient populations	Develop intentional systemic ways to diversify patient populations
Lack of insurance and access to care	Engage multidisciplinary teams to minimize barriers to care
Lack of transportation	Increase virtual visit opportunities Subsidize travel or offer transportation Partner with clinics closer to patients
Systemic barriers	
Ineffective programs for community outreach and patient recruitment	Increase and diversify community outreach and advocacy programs Diversify approaches to patient recruitment early in the process
Mistrust of health care and research	Create opportunities that build and enhance trust
Restrictive eligibility criteria	Systematically examine and modify eligibility criteria
Personal barriers	
Low socioeconomic status and financial concerns	Increase funding for minority and financially disadvantaged patients Incentivize trial participation Simplify reimbursement and remuneration strategies

Perceived logistical barriers to URM patient recruitment in cancer clinical trials were lack of workforce diversity and the underutilization of patient navigators (i.e., people who help patients navigate the health care system). This was demonstrated by the shortage of diversified staff who could potentially enhance cultural concordance, and by overall limited staffing. Additional logistical barriers reported were restricted access to diverse patient populations, lack of insurance, and lack of access to care or transportation. Perceived systemic barriers to URM patient recruitment included ineffective community outreach and patient recruitment programs that resulted in the exclusion of out-of-network patients, mistrust of health care and research, and restrictive clinical trial eligibility criteria. Eligibility criteria for a clinical trial can be influenced by regulatory bodies with differing standards or the presence of a diagnosis or disease that may disproportionately preclude URM patients from participating in clinical trials. Finally, perceived personal barriers to URM patient recruitment included low socioeconomic status and financial concerns.

The corresponding strategies suggested to improve the recruitment of URM patients in cancer clinical trials included accelerated intentional efforts to address workforce diversity gaps, such as increasing staffing and creating designated roles for patient navigators who can promote cultural concordance and directly recruit patients for clinical trials (Table 2). Other suggested strategies were increasing and diversifying community outreach and advocacy early in the recruitment process, partnering with clinics closer to patients, creating opportunities that build and enhance trust, assisting URM patients with logistical and transportation needs, and systematically examining and modifying eligibility criteria. In the software-assisted analysis of participant responses, thematic saturation was reached, defined as “no new ideas or themes emerging.”

## Discussion

Our study provides unique insight into the perspectives of health care professionals on barriers to URM patient recruitment in cancer clinical trials and strategies that may help overcome those barriers. Unlike in previous studies, the participants in our study were representative of different types of health care professionals including physicians and clinical research staff, which makes this study one of the most extensive.

Notably, several study participants described the need for increased workforce diversity to improve the recruitment of URM patients in cancer clinical trials. The importance of workforce diversity in clinical trials cannot be overstated. Diverse teams offer a broad range of experiences, perspectives, and skills that can foster creativity, innovation, and better problem-solving abilities.^14^ Staff diversity can also facilitate the recruitment of URM patients by helping to overcome unconscious bias, stereotyping, and cultural misunderstandings that may hinder the establishment of trustful relationships with URM patients.^15^

When considering workforce diversity, studies have demonstrated the importance of clinical research nurses (CRNs).^14–16^ Given that CRNs have direct, regular contact with patients, they have the opportunity to act as patient advocates, educators, and advisors.^14^ CRNs can also bridge cultural and linguistic gaps, explain medical jargon, and offer culturally sensitive care—all of which can considerably enhance patient comfort, trust, and willingness to participate in trials.^14–16^ Moreover, CRNs of similar ethnicity to URM patients can foster a sense of belonging and acceptance, making patients feel more understood and valued. Thus, systematically addressing workforce diversity gaps by strategically incorporating diverse CRNs and clinical research staff overall could substantially enhance URM patient recruitment in clinical trials. The findings of our study support those of previous studies^17,18^ showing the importance of workforce diversity and the use of patient navigators.

Our study participants also described low socioeconomic status and lack of insurance coverage, access to care, and transportation as barriers to the enrollment of URM patients in clinical trials. Similarly, other studies have identified socioeconomic factors and insurance coverage as major determinants of access to clinical trials.^19^ Lower socioeconomic status often correlates with poorer health literacy, reduced access to health care services, and greater financial constraints.^20^ People from low socioeconomic backgrounds may lack the resources or time to participate in trials, or they may face more substantial barriers to accessing care in the setting of a trial.^19,20^ Inadequate insurance can further aggravate these disparities, limiting access to health care professionals or facilities that offer clinical trials.

Another concern raised by our study participants was restricted access to diverse populations due to the less diverse communities served by tertiary care institutions. Multifaceted solutions are needed to address this issue. First, designing a community-centric outreach initiative that helps disseminate easy-to-understand information may increase awareness and reduce misconceptions about clinical trials. Second, tailoring recruitment approaches to low socioeconomic circumstances, such as providing financial support, flexible trial schedules, or transportation assistance, may increase participation. Finally, at a policy level, negotiating with health insurers and industry sponsors of clinical research could improve insurance coverage for trial-related costs and improve access to care for all patient populations.

Ineffective community outreach and patient recruitment, as well as mistrust in health care and research, were also described by our study participants as considerable barriers to URM patient recruitment. Language barriers and the use of complex medical jargon greatly impede URM patient recruitment and retention in clinical trials. Potential participants may feel overwhelmed, confused, or intimidated by the technical language used in consent forms and informational materials.^3,21–23^ Consequently, patients may hesitate to participate or, in the worst-case scenario, provide misinformed consent. Addressing language barriers involves simplifying the language used in patient-facing materials and providing translations in languages relevant to the target population. In addition, comprehension can be improved by applying health literacy principles to create easy-to-understand informational materials and consent forms. Engaging bilingual staff or interpreters during patient interactions can also foster understanding, build trust, and encourage participation.^23^ Moreover, rethinking community outreach and building sustainable, genuine relationships with communities to engage people early in the research process could strengthen URM patient recruitment.

As described previously,^24^ our study participants reported that the geographic location of clinical trial sites can substantially affect URM patient recruitment. Many clinical trials are conducted at urban tertiary care centers, which may present challenges for people residing in rural or remote areas. This geographic disparity can inadvertently exclude potential URM participants, reducing the diversity of trial enrollees.^24–28^ Strategies to combat geographic disparities could involve decentralizing trial locations, such as offering trials in community hospitals, satellite clinics, or through telemedicine if possible. Offering trials at other campuses or institutions with larger URM patient populations is another way to increase the accessibility of trials to a wider demographic. This would not only enhance the diversity of trial participants but also increase the generalizability of the study findings.^3,14–16,19–30^ Finally, designing clinical trials that have less restrictive eligibility criteria was identified in our study as a strategy to enhance the recruitment of URM patients. Previously, standard clinical trial eligibility criteria were shown to disproportionately exclude Black patients with cancer compared with White patients with cancer.^31^

The major limitations of this study include our single-institution design and our overall low rate of URM patients treated for cancer. In addition, most of the health care professionals and clinical research staff interviewed in our study were White, potentially introducing bias. Nevertheless, although our study was conducted at a single tertiary academic institution, our findings resonate with those of broader academic clinical research centers.^32^ In addition, the recommended strategies highlighted in our study are consistent with those described in the literature and provide potential pathways to enhance URM patient recruitment in clinical trials. Despite the limitations of our study, the findings underscore the need for a comprehensive, multifaceted approach to addressing barriers to recruiting URM patients into clinical trials. The continual process of evaluating and implementing solutions and strategies is paramount for enhancing URM patient recruitment and care and will ultimately promote cancer research that benefits diverse populations.

## Conclusion

In this study, we comprehensively explored the challenges and opportunities encountered in recruiting URM patients for therapeutic cancer clinical trials. The strength of our research lies in its breadth, capturing the perspective of a range of health care professionals involved in patient care and clinical trial recruitment. The insights presented here reveal the intersection of personal, systemic, and logistical barriers that require nuanced approaches to overcome. The recommended approaches from our research participants highlight the need for a comprehensive and continually evolving strategy for addressing these barriers. Future studies are warranted in which the proposed strategies are systematically implemented and evaluated.

## Data Availability

All relevant data supporting the findings of this study are reported within the article or are available from the corresponding author upon reasonable request.

## Abbreviations Used

CRNs: Clinical Research Nurses
URM: Under-Represented Minority
