# Supplementary material for: Qualitative Study of Health Care Professional Perspectives on Recruiting Minority Patients in Cancer Trials
Source: Health Equity. 2025 Jan 20;9(1):53–9. doi: 10.1089/heq.2024.0184 (PMC11848063; doi:10.1089/heq.2024.0184)
Supplement: Supplementary Data [file heq.2024.0184_supplementarydata.docx]

**CLINICIAN AND STAFF INTERVIEW GUIDE**

**Introduction**: Thank you for agreeing to participate in this study. I’d like to talk with you about your involvement in the care process of underrepresented minority (URM) patients who are enrolled in clinical trial at Mayo Clinic. We are talking with patients, too; but in this interview, we hope to learn about some of the strategies for recruitment, challenges and facilitators for recruiting patients, and your overall perspectives on how the care and recruitment process can be improved for URM patients on cancer trials; specifically, for this study, we are interested in learning about interventional cancer treatment trials.

1. Can you describe your specific role in the care of URM patients who are on clinical trials?
   1. What role do you play in the recruitment of patients in interventional cancer clinical trials?
   2. Thinking about the tasks you perform to care for these patients, are there certain procedures/processes that you need to follow to accomplish those tasks?
2. Let’s talk about some of the strategies that you have historically used for recruitment of URM patients to interventional cancer clinical trials.
   1. What are the strategies?
3. Can you share some of the challenges you face in recruiting URM patients in a trial?
   1. What about challenges you face in the care of URM patients in trials?
4. Are there specific strategies that work better than others in recruiting URM patients? Explain.
   1. Alternatively, are there strategies that do not work? Explain.
5. With regard to the processes you have historically used, are there key aspects that need to be addressed or areas that can be enhanced to increase URM patient accrual to clinical trials? What are those, and how can they be addressed?

©2024 Mayo Foundation for Medical Education and Research.
