# Supplementary material for: Qualitative Study of Health Care Professional Perspectives on Recruiting Minority Patients in Cancer Trials
Source: Health Equity. 2025 Jan 20;9(1):53–9. doi: 10.1089/heq.2024.0184 (PMC11848063; doi:10.1089/heq.2024.0184)
Supplement: Supplementary Table S1 [file heq.2024.0184_supplementary_tables1.docx]

**Supplemental Table.** Key Themes from Interviews of Healthcare Professionals and Clinical Research Staff on Challenges and Barriers to Underrepresented Minority Patient Recruitment to Cancer Clinical Trials

| **Challenges and barriers** | **Example quotes** |
| --- | --- |
| **Lack of workforce diversity**  *Staff not reflective of diverse communities being served*  *Lack of cultural concordance* | “But I think even then, being aware of the population you're trying to reach, if it is, say, the Hispanic population, having somebody who is speaking that language or from that community I think would be beneficial to the recruitment. Something like that, speaking the language to make it more comfortable for that person to ask the questions they need to and feel like they get a good, complete answer from that person.” **CRC Supervisor**  “So, for many years we didn't have any minority representatives … we're doing better now. We have more people, and it's more African American…We don't have any Native-American or... I think it's always going to be somewhat limited because you can't be exactly representative, but I think at least having a diversity of faces…might be reassuring that it's a priority. And so, I think that's one part of it, is making sure the team itself is diverse because everybody can then hear and understand some of the issues that may not be immediately obvious.” **MD**  “I assume there will be, so let's say a Somalian guy, right? I mean, he or she will be much more open to talk about a research study if there is someone who is also from Somalia talking to them about this research, right? So maybe a clinical coordinator who is from Somalia and then understand and can speak in their language and things like that.” **Scientist**  “And I think having minority patients [to] see minority providers is very important for them to see that representation and the staff that's taking care of them.” **PA** |
| **Underutilization of patient navigators**  *Short staffing* | “So I think that this is a good strategy that we have a navigator role to help increase the minority accrual population…her role is still evolving and will be, but I think that's probably the most strategic move right now is to go through these charts and show that there are 82 black women that came into the breast clinic last week. Why were none of them talked to about research trials?” **RN Navigator**  “Recommend designated staff CRCs who would regularly filter provider schedules and then prescreen the identified patients for any available trials open to that specific pod or disease group.” **RN Navigator**  “And so I think the biggest thing, honestly, for clinical trials, instead of trying to get minorities to Rochester for them would be doing the clinical trials in a place they're already at….So significantly shorter on the staffing part of it and to try and boost up more clinical trials would be great, but you got to staff for it and train that staffing as well.” **PA** |
| **Ineffective community outreach and patient recruitment**  *Out-of-network recruitment* | “There are certain studies that we would do that screening for, that we would just go directly out into the community. Where we would actually go into the community and make it just easier to reach the community individuals. Because even just where Mayo Clinic Florida is located… it's actually really out of the way for a lot of people.” **CRC**  “Getting community participants to advocate for the research that's being done…getting enough people who are the community leaders to say that this is something that the community should do? I don't know that that's being done enough, and I think that might be even more important than really us going out there.” **CRC Supervisor**  “We try and do community outreach and those kinds of approaches, because I think in some ways, we have to go to people rather than having people come to us.” **MD**  “And it might be interesting to see what happens if we did accept patients that were out of network with us and things. I do know that those barriers exist outside of the hospital and that sometimes just getting care, as these patients go to multiple ERs and see multiple providers, can be tough.” **PA**  “The concept is that we're supposed to approach people equally, like we're not supposed to alter the approach of a clinical trial because we're trying to be more equal, but you have to acknowledge that it's different. Certain groups of patients are going to have more hesitancy than others just based on history, and very justified, rational, understandable hesitation, and if you approach equally the same way to everyone, that's actually not, that's actually still putting groups at a disadvantage… about just because focusing on making everything the same is always going to be problematic towards the group of people who are already more hesitant. You don't need to convey why it's a good idea to a group that's never been treated poorly with clinical trials.” **CRC**  “There are not strong community engagement programs to patients around, or even Mayo situation itself, within the community that patients feel comfortable, especially if they are from low income or socioeconomic status to even come to Mayo.” **MD**  “Sometimes I've reached out to local providers, I've reached out to colleagues who are at other academic institutions. If we have studies that they don't have, or they have studies we don't have, we want to get the patients to the best place.” **MD** |
| **Restricted access to diverse patient populations** | “Try to involve more the centers in Florida and Arizona. Because just as I said, the same reason that in Minnesota, most people here are white. In those two other states, maybe the population is more diverse, right? At least more in the Latino part.” **Scientist**  “I think barrier number one is, you have to see minority patients, to be honest before you can enroll them, and we just don't see enough unfortunately.” **MD**  “So, the people I see are already screened in many ways that have nothing to do with anything I can control. So, it's screened based on insurance, and demographics, and where they live, and access they have to our institution and all of that. So right from before I even meet the patient there is many things that go into determining who I even see in the first place. And that's where the fundamental biggest problem is in my mind. If I'm seeing 95% Caucasian patients, then I can't put 20% minority patients on clinical trials, because it's just not the type of patients that I see …That's more an institutional issue as to what types of patients are we looking at.” **MD**  “The start point is changing the fundamental question of, are we willing to accept more diversity in our clinical trials process at the expense of losing money? Because ultimately, it's an investment in the future and in equality and diversity.” **MD** |
| **Insurance and access to care** | “There are other ways to get around the insurance problem, but a coordinator might face, ‘Well, we couldn't bring them in because they didn't have the right kind of insurance or an access problem.’” **CRC**  “It all comes back to money and insurance. Again, a lot of people assimilate research with free care. So, there is the need for them to be explained that ‘yes, you might be eligible for this clinical trial, there are going to of be some charges to you.’ So that's where you have to reiterate the insurance is important because I get this statement all the time. ‘Well, if I'm volunteering to be on your research trial, then I shouldn't have to pay anything.’” **RN Navigator**  “Some days truly because I get some really sad stories or people, when I read their history, it's like, you truly want to help them. And I listen and I can talk and be empathetic and whatever. And then at some point ‘this is as far as I can go helping you, you now need to call this phone number to see if you're going to be able to come in.’ And so that's the biggest hindrance or the obstacle there.” **RN** **Navigator** |
| **Transportation and logistics**  *Distance to and from clinic* | “The travel was always a big one and I think that stands out for us. Our sheer location, we are just not possible to a downtown area or a place where a bus schedule would go to.” **CRC**  “We are not in a major metropolitan area. Why do I say that? Let's say we're targeting a Native American population for a trial and the nearest high population density is 200 miles away. That is an obstacle because now we have to include travel, more travel, more loss of work, more childcare. That's why we really, I think, need to do partnerships with other places that may be closer to these geographic centers. We're still in a cornfield down here. It's a great cornfield and we do a lot of things.” **MD**  “Well, some of our trials require long term therapy and its often hospital based. They need to be here in Rochester for weeks, months, sometimes many months. Some of the clinical trials involve several years of therapy. It becomes challenging given our kind of our geographic area and they're coming from far away. To be away from home at a long period of time is a challenge… Sometimes they'll decline, not because they're not interested in the trial, but because they can't commit to being here in Rochester.” **MD** |
| **Low socioeconomic status/financial issues** | “I think we're not talking that much as race as a socioeconomic status. That usually goes towards, more incomes tend to be white, and less incomes tend to be other races. So, for the same reason, people who get access to Mayo usually are more in the white side.” **Scientist**  “So, there either needs to be maybe designated funding for underrepresented populations that could share the burden of the financial aspect of coming to Mayo Clinic, or accepting more, expanding the insurance acceptance library, for lack of better verbiage.” **RN Navigator**  “I know that some clinical trials will reimburse travel or lodging, and I think that’s really helpful. Some will even pay patients. I think that added incentive is nice for patients because they're giving a lot of their time and not necessarily always benefiting from the radiology clinical trial; they're not benefiting from that, they're just doing it to carry science forward.” **PA**  “A lot of trials do reimbursements, but that requires fronting the money. It is a reimbursement, and there are a lot of hoops to jump through to actually get that money… For example, we have to have a copy of the receipt of everything they would reimburse, and they have to be able to read it. It has to meet certain standards.” **CRC**  “There are issues with all groups in terms of the financial toxicities of the treatments cancer treatment is, and that's a real problem because we see it in patients who are not able to get the treatment they need. Or they come, and then they have to leave us because they have too much debt in which they're not able to pay.” **MD**  “And then sometimes, too, more so frequently the insurance products that are being offered have a significant out of pocket expense which is unaffordable for many patients.” **MD**  “I see this as a socioeconomic issue. I see it as a problem for middle class dual income people that both have to work, to keep their house, to put food, to pay for their mortgage. Unfortunately, maybe it bears out... and ethnicities and minorities bearing the brunt, because maybe they comprise more.” **MD**  “That's one thing we are doing. But as you know, even that's not enough. We, as an institution should begin to consider, can we negotiate contracts with health care insurers… we will discount the care, we will work with the clinical trials sponsor to say, we will help with travel vouchers, we will help with mileage reimbursements.” **MD** |
| **Mistrust of research and health care** | “But then the other thing, is that also I think this is also about cultural things, in cultural barriers. I heard that the, for example, African American population, actually they usually tend to distrust a little bit more on clinical care. Again, probably because of history that I can't blame them about the things like that. So sometimes it's not only about getting them to come to Mayo, but also to try to go across that barrier.” **Scientist**  “Some people's opinions for clinical trials are the same as anti-vaxxers. ‘You're not putting anything in my body that hasn't been around and been proven. You mean you want me to be a Guinea pig? I mean, that's...’ And then that can be addressed. So, the patient's level of understanding.” **MD**  “Sometimes we do see in individual patients the lack of trust in the system. Usually by the time they are within our system, and they are under my care and receiving the care, it's a free country so people can leave and seek other providers. Or if they're not happy, they do that and sometimes we have that with all racial and ethnic groups, not just minorities. And by the time they have been under my care for quite a long time then the trust is there and there's really no major issues.” **MD** |
| **Eligibility criteria**  *Regulatory issues*  *Regulatory bodies comprising different standards*  *Diagnosis and disease factors* | “And I've found that they're not put on trial either because there isn't progression, they have stable disease, or they're already on a trial, or the eligibility criteria, they just don't fit. A lot of our trials right now are so focused on genetic mutations, and you have to have so many different treatments. It makes it really tough to get into these trials.” **RN Navigator**  “There's been times where I've had a patient who needs a phase one, but they open and close every single day because there's so many different sites around the country and they're only allowing four or five patients into that. So, it happens that they need the trial, they want the trial, but we don't have an open. And then, there's times where I'm screening and I'm finding patients for studies that we have, but they're just not open yet.” **RN Navigator**  “So, their time to diagnosis is longer. And so that means their disease has progressed more. And so, we have to understand that that disease is going to affect their organs more, which means they're not going to be as good. And so having a trial say your organs have to be this good is not fair because they do not have the same opportunity for diagnosis.” **PA**  “Then, in our world, melanoma, there is a lot of metastases to the brain and that is often a disqualifier for clinical trials. That's a common frustration for me.” **PA**  “We have a lot of rules in place about who can and who can't come in the door, and who's green-lighted and who's red light and who's yellow-lighted. And so, all of that determines really who I personally see in the end of the day and then can offer a trial too.” **MD**  “That's a challenge because they have so many side effects from the treatment that it's part of what's expected with the treatment, but then we have to report them all. The regulatory burden of clinical trials has definitely gotten more challenging over the years. Made it more difficult for us. It's more time-consuming for us to do those things.” **MD**  “And so having certain organ function optimizations may really limit the amount of minorities because we just know historically their time to diagnosis is going to be a little bit longer and which means their disease is going to have more time to progress. So, thinking about that, are there certain ways that we could say, you know, what if this person, you know, looking at their history, was seen repetitively for the same problem; can we go ahead and omit these markers? That way, they can still be on trial?” **PA**  “If you feel like their disease is at a point where if this clinical trial could help them, get nephrology on board and analyze them. You know, optimize the kidneys as much as you can. And if you need to, you can do a temporary dialysis and get them there. Same with a heart patient, get cardiology on board, say, ‘how can we optimize this?’” **PA**  “A big thing that they can do, if they're interested in helping make these trial requirements more flexible, is reach out to some of these big pharma, medical science liaisons, and people who always want to talk to these consultants. Let them know, ‘Hey, we want more minority patients. We want to increase our accrual of them. Hey, this is what we think will help,’ and then start working with them. Like this is a team effort, you know? The clinical trials come from big pharma. Have some of these consultants who are key opinion leaders in the community reach out and say, ‘Hey, this is what we're thinking. Because if these doctors are saying this, that's probably going to be the best thing to help it change.’” **PA** |

Abbreviations: CRC, clinical research nurse; MD, medical doctor; PA, physician assistant; RN, registered nurse; URM, underrepresented minority
